# Engaging with nature and work: associations among the built and natural environment, experiences outside, and job engagement and creativity

**DOI:** 10.3389/fpsyg.2023.1268962

**Published:** 2024-01-11

**Authors:** Rebecca M. Brossoit, Tori L. Crain, Jordyn J. Leslie, Gwenith G. Fisher, Aaron M. Eakman

**Affiliations:** ^1^Department of Psychology, Colorado State University, Fort Collins, CO, United States; ^2^Department of Psychology, Louisiana State University, Baton Rouge, LA, United States; ^3^Department of Psychology, Portland State University, Portland, OR, United States; ^4^Colorado School of Public Health, Aurora, CO, United States; ^5^Department of Occupational Therapy, Colorado State University, Fort Collins, CO, United States

**Keywords:** built environment, natural environment, outdoor experience, outdoor activities, job engagement, creativity

## Abstract

**Introduction:**

There is substantial evidence that contact with nature is related to positive health and well-being outcomes, but extensions of this research to work-related outcomes is sparse. Some organizations are redesigning workspaces to incorporate nature and adopting nature-related policies, warranting a need for empirical studies that test the influence of nature on employee outcomes.

**Methods:**

The present mixed-methods study tests and extends the biophilic work design model to examine associations among the built and natural environment at work and home, experiences of time spent outside (i.e., amount of time outside, enjoyment of time outside, outdoor activities), and motivational work outcomes (i.e., job engagement and creativity). Objective geographic data were combined with quantitative and qualitative survey responses from working adults (*N* = 803).

**Results:**

Our results broadly indicate that individuals who work and live in areas with greater natural amenities (i.e., access to water, topographic variation, temperate climates) spend more time outside and enjoy time outside to a greater degree, and these experiences are in turn associated with greater engagement and creativity at work. We did not find evidence that the surrounding built environment (i.e., urbanity) at work or home was associated with outdoor experiences or work-related outcomes. Additionally, six categories of outdoor activities were identified in the qualitative analyses – leisure activities, relaxation, physical activities, social interactions, tasks and errands, and travel.

**Discussion:**

The findings from this study provide evidence that the natural environment, particularly at home, can benefit work-related outcomes via greater time and enjoyment of time outside. This study has implications for employee time use and organizational effectiveness.

## 1 Introduction

Spending time outside in nature can have a positive influence on human health, well-being, and cognitive functioning (Kaplan and Berman, 2010; MacKerron and Mourato, 2013; Hartig et al., 2014; McMahan and Estes, 2015; Twohig-Bennett and Jones, 2018; Yao et al., 2021). Unsurprisingly, companies are responding to these developments and incorporating nature into the physical design of the work environment and in employee wellness policies and programs. For example, Apple, Facebook, Amazon, and Walmart redesigned their headquarters to incorporate more natural features, such as nearby meadows, lakes, trails, rooftop gardens, and indoor plants (e.g., Sears, 2016; Mafi, 2017; Kwun, 2018; Wilson, 2019; Klotz and Bolino, 2021). Other companies have nature-related policies – Patagonia has a *Let My People Go Surfing* policy, in which employees are encouraged to go outside during the workday (Chouinard, 2006); Recreational Equipment, Inc. (REI) employees get two paid *Yay Days* a year, during which they are expected to spend time outdoors and reconnect with nature (Recreational Equipment, Inc., n.d.). Thus, it appears organizational leaders are inferring that the wide-ranging benefits of nature will also translate to employee and organizational effectiveness. However, few studies to date have examined links between nature and work-related outcomes.

Despite the theoretical and empirical groundwork suggesting that the benefits of nature should extend to work-related outcomes (e.g., Kaplan, 1993; Klotz and Bolino, 2021), research in this area is limited. Job engagement and creativity at work are two particularly important work-related outcomes, as both are positively associated with employee job performance and provide a competitive advantage to organizations (e.g., Christian et al., 2011; Zhou and Hoever, 2014; Brown, 2016; Madsen, 2017). Accordingly, successful companies (e.g., Apple, Southwest Airlines, Ben & Jerry’s, Google) are often recognized for having employees who are highly engaged and creative (Chitre and Buss, 2013; Phelps, 2019; Ang, 2020). Although experiences of job engagement and creativity are related to employee well-being (Halbesleben, 2010; Mumford and Todd, 2019), job engagement and creativity are conceptually distinct from more commonly studied health and well-being outcomes. Instead, engagement and creativity are motivational work-related constructs that rely on having available emotional, cognitive, and physical energy resources (Amabile et al., 2005; Atwater and Carmeli, 2009; Rich et al., 2010; Zhou and Shalley, 2011); that are afforded by contact with nature (Klotz and Bolino, 2021). In this way, the benefits of nature may transcend more traditional assessments of health and well-being and have a positive influence on work-related outcomes of job engagement and creativity.

### 1.1 Theoretical framework: biophilic work design model

The biophilic work design model applies Kaplan and Kaplan’s (1989) seminal attention restoration theory to workplace settings to explain how contact with nature at work can have energizing effects on employees. By integrating theories of human energy (e.g., Kruglanski et al., 2012; Quinn et al., 2012), with research on nature’s impact on emotional, cognitive, physical, and prosocial outcomes (e.g., Keniger et al., 2013), Klotz and Bolino (2021) identify how greater contact with nature at work can restore employees’ *potential* energies; resources that can be stored and drawn upon in the future (Quinn et al., 2012). Specifically, *emotional potential energy* supports positive feelings (e.g., enthusiasm) and helps employees regulate their emotions, *cognitive potential energy* allows employees to regulate their thoughts and maintain directed attention, and *physical potential energy* enables feelings of health and strength. Each of these forms of potential energy can be replenished by contact with nature (e.g., Kaplan and Berman, 2010; Hartig et al., 2014; McMahan and Estes, 2015; de Keijzer et al., 2016; Twohig-Bennett and Jones, 2018).

However, Klotz and Bolino (2021) focus on energies and do not identify specific work-related outcomes in their theory. Therefore, we extend the biophilic work design model by examining job engagement and creativity at work. *Job engagement* is defined as a positive motivational state characterized by the exertion of emotional, cognitive, and physical energy in one’s work role (Rich et al., 2010). *Emotional engagement* is characterized by high pleasantness and activation, including feelings of positivity, enthusiasm, and interest in one’s job. *Cognitive engagement* reflects focus and concentration in one’s job. *Physical engagement* is defined as working with intensity and exerting energy in one’s job. Next, *creativity* is defined as the generation of new and useful ideas, approaches, or solutions (e.g., Hennessey and Amabile, 2010; Anderson et al., 2014; Mumford and Todd, 2019). Like engagement, creativity at work is considered a motivational construct (e.g., Amabile, 1997; Zhou and Shalley, 2011) and relies on available emotional, cognitive, and physical energies to perform job tasks that may require or benefit from creativity. For instance, positive affect and emotions are conducive to creativity at work (e.g., Amabile et al., 2005; George and Zhou, 2007), being creative is an inherently cognitive process that requires higher-order cognition (e.g., Dietrich, 2004), and feeling energized at work is associated with workplace creativity (e.g., Atwater and Carmeli, 2009). In this way, energies gained by exposure to nature should enhance employees’ job engagement and creativity at work.

Additionally, as another extension of the biophilic work design model, we consider the built and natural environment in both the work and nonwork (i.e., home) domain. Specifically, the type of outdoor environment where individuals work *and* live are explored, and their experiences of time spent outside during work *and* nonwork time are examined. Ultimately, the replenishment of energies may be different across these unique settings, resulting in different practical implications about where, when, and how time should be spent outside to foster engagement and creativity at work.

### 1.2 The present study

We explored associations among the built and natural environment where individuals work and live, experiences of being outside, and work-related outcomes. The limited studies on nature in relation to job engagement and creativity have either exclusively focused on the indoor work environment (e.g., office plants, window views, videoconferencing backgrounds) or examined time spent outside more generally, rather than considering the surrounding built and natural environments (Bringslimark et al., 2007; Nieuwenhuis et al., 2014; Plambech and Van Den Bosch, 2015; Korpela et al., 2017a,b; Hyvönen et al., 2018; Thompson and Bruk-Lee, 2019; Hähn et al., 2020; Fleury et al., 2021; Petersson Troije et al., 2021; Palanica and Fossat, 2022). Consequently, researchers have called for studies that assess more nuanced aspects of nature, such as varying degrees of urbanity and naturalness, proximity to water or topography, and actual use of greenspaces (e.g., Keniger et al., 2013; Hartig et al., 2014; Bratman et al., 2015; de Keijzer et al., 2016). In response to these recommendations, we conceptualized the outdoor environment in terms of the surrounding urbanity and natural amenities. *Urbanity* reflects the population size and density of an area. *Natural amenities* are geographic and weather-related characteristics typically considered as desirable, including moderate temperatures, low humidity, topographic variation (e.g., mountains), and bodies of water (McGranahan, 1999).

Further, we explored three aspects of the *experience* of being outside – amount of time spent outside, enjoyment of time spent outside, and outdoor activities. We used a concurrent mixed-methods approach, in which quantitative data (i.e., objective geographic data and survey responses) and qualitative data (i.e., responses to open-ended survey questions) were collected at the same time, and each used in the interpretation of the results (Clark and Creswell, 2008; Pluye and Hong, 2014). Our primary goal of the study was to understand how aspects of the built and natural environment, and outdoor experiences, are associated with employee work outcomes. To answer this question, quantitative data were used to demonstrate features of the built and natural environment, elucidate how much time employees spend outside during work and nonwork time, and identify the extent to which they enjoy the time they spend outside. Analyses of the qualitative data were used to classify the specific activities in which individuals engaged while they were outside. Findings from the qualitative responses therefore complement the primary research question assessed via quantitative surveys by providing more nuanced information about how participants actually spent time outside, thus capturing a unique and important piece of the outdoor experience. This qualitative approach contrasts with existing work on outdoor activities which typically uses researcher-generated categories in a checklist format, which may not include all types of activities individuals engage in outside. Instead, asking participants how they spent their time outside yields more specific and accurate information that is less influenced by potential researcher bias (i.e., assumptions about how individuals spend their time outside) than if pre-determined categories were used, highlighting an advantage of mixed-methods approaches (e.g., Mazzola et al., 2011). Overall, in combination with the quantitative model, the qualitative data were used to identify the various outdoor activities individuals performed at work and at home, whether activities were comparable or different in these distinct domains, and how activities were associated with work outcomes.

### 1.3 Hypothesized effects

#### 1.3.1 The role of the built and natural environment on engagement and creativity at work

First, we expected that individuals who work and live in less urban areas and places with more natural amenities should experience greater contact with nature and be more engaged and creative at work. Natural environments are considered highly restorative (Kaplan and Kaplan, 1989; Menardo et al., 2021; Yao et al., 2021), and much of what is known about the benefits of nature has come from studies that detected benefits after exposure to natural but not urban settings (e.g., Berman et al., 2008; White et al., 2013; Bratman et al., 2015; McMahan and Estes, 2015; Pilotti et al., 2015). Related research has demonstrated that contact with nature – through indoor environments or time spent outside – is linked with greater engagement and creativity at work (e.g., Hyvönen et al., 2018; Thompson and Bruk-Lee, 2019; Petersson Troije et al., 2021; Bergefurt et al., 2022; Palanica and Fossat, 2022), though most of this past work has not explored the degree of surrounding urbanity or natural amenities.

#### 1.3.2 The role of the built and natural environment on experiences of being outside

Individuals who work and live in areas that are less urban, and afford greater natural amenities, will likely *spend more time outside* (Hartig et al., 2014). Inaccessible natural spaces, safety concerns, perceived lack of fresh air, overdevelopment, and crowdedness can prevent urban residents from spending time outside (Kellert et al., 2017). Conversely, a person’s inclination to spend time outside should be greater when a location has natural amenities, like mountains and access to water, which can provide varied activity options (e.g., wildlife photography, swimming; Georgiou et al., 2021). Moreover, weather can prevent people from spending time outside as well (Kellert et al., 2017), so natural amenities like low humidity and temperate climates should be associated with more time spent outside. For similar reasons, individuals should also *enjoy time outside* to a greater degree when they are in less urban places and locations with more natural amenities. The idea that natural environments are inherently enjoyable and preferred over urban settings has gained some empirical support (e.g., Herzog et al., 2003; Kaplan, 2007; Van den Berg et al., 2014; Klotz, 2020). Indeed, natural settings, particularly those with water, are commonly cited as favorite places (Korpela et al., 2001; White et al., 2010).

#### 1.3.3 The role of outdoor experiences on engagement and creativity at work

Engagement and creativity at work may be enhanced by spending more time outside and enjoying time spent outside. Spending time outside provides the greatest contact with nature and enables the replenishment of emotional, cognitive, and physical energy stores (Kaplan and Kaplan, 1989; Klotz and Bolino, 2021). Regarding enjoyment of time spent outside, compatibility between an environment and an individual’s preferences is a critical feature of whether an environment will effectively restore energy (Kaplan and Kaplan, 1989). Past work has also demonstrated that being outside more during work and leisure time is associated with greater effort and engagement at work (Hyvönen et al., 2018; Bergefurt et al., 2022; Klotz et al., 2022). One qualitative study examined the experiences of office employees in Sweden who brought their work tasks outside and found it was common for participants to report that working outdoors enhanced their ability to concentrate, think through and solve problems, and feel more inspired and creative at work (Petersson Troije et al., 2021). In addition, enjoyment during work breaks and leisure time is associated with favorable emotional, cognitive, and physical outcomes, like positive emotions, better concentration, and reduced health complaints and fatigue (e.g., Trougakos et al., 2008; Van Hooff et al., 2011; Newman, 2014; Hunter and Wu, 2016; Sianoja et al., 2018). Furthermore, enjoyment has been conceptualized as a feature of the experience of recovering from work and possible work-related stressors. Employees who are able to recover from work stress are often more engaged and creative workers (e.g., de Jonge et al., 2012; Sonnentag et al., 2012, 2017; Sianoja et al., 2018).

#### 1.3.4 Hypotheses

**Figure 1:**
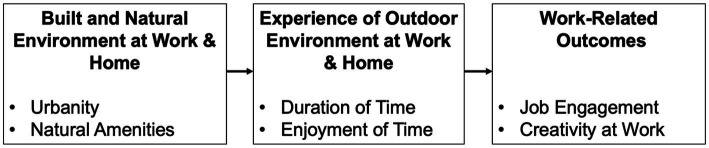
Conceptual model.

When individuals who work and live in urban environments spend time outside, they are more likely to be exposed to distractions (e.g., traffic, advertisements, crowds) and other barriers that can prevent them from wanting to stay outside, enjoying their time outside, and ultimately reaping the benefits of being outdoors. On the other hand, those who work and live in areas with more natural amenities should spend more time outside, find time outside more enjoyable, and experience the restorative benefits of nature. In turn, these individuals should acquire emotional, cognitive, and physical energies, resulting in a greater ability to devote attention to their job, feel energetic in their job, work with intensity, and generate new and innovative ideas (see Figure 1).

*Hypothesis 1*: The negative associations between the urbanity where individuals work and live and their engagement and creativity at work will be mediated by (a) *less time* spent outside and (b) *lower enjoyment* of time spent outside.

*Hypothesis 2:* The positive associations between the natural amenities where individuals work and live and their engagement and creativity at work will be mediated by (a) *greater time* spent outside and (b) *greater enjoyment* of time spent outside.

### 1.4 Exploratory research questions

Beyond the amount of time spent outside and the enjoyment of time spent outside, *how* people spend their time outside is another important feature of their experience outdoors. In past work, researchers have focused on specific types of outdoor activities, typically physical activities, such as exercising outside (Barton and Pretty, 2010) or walking in a park (e.g., Sianoja et al., 2018). Others have explored a wider range of activities individuals engage in outside, but very little work has examined outdoor activities during work time (e.g., Petersson Troije et al., 2021), or combined both work and nonwork time use (e.g., Lottrup et al., 2012; Korpela et al., 2014; Hyvönen et al., 2018). Stigsdotter and Grahn’s (2011) study included a comprehensive list of outdoor activities, but they explored which activities were preferred by Swedish individuals who experienced high levels of stress. Kellert et al. (2017) indicated that walking and hiking are American adults’ favorite outdoor activities, but did not examine the specific outdoor activities in which individuals engaged, nor did they consider activities during both work and nonwork time. Further, despite substantial research on general time-use (e.g., Stinson, 1999), less is known about outdoor-specific activities. Overall, it is unclear how individuals spend time outside during work time, and how this compares to their nonwork time spent outside.

*Research Question 1:* In which activities do individuals participate when they are outside during work and nonwork time? How are outdoor activities during work and nonwork time similar or different?

Certain types of outdoor activities may be associated with favorable work outcomes. Oppezzo and Schwartz’s (2014) four-part study found that walking has a positive effect on general creative thinking, and that walking outside, compared to indoor environments, is particularly beneficial for idea generation. Other research suggests that walking outside can improve concentration at work, as well as general cognitive performance outcomes and mood (e.g., Berman et al., 2008; Sianoja et al., 2018), factors that can influence job engagement and creativity. White et al. (2013) found that participants who visited nature reported feelings of restoration when walking, and that nature visits with children were less restorative than visiting nature alone. Psychological benefits have also been found for other types of outdoor physical activities (e.g., swimming, running, cycling; Barton and Pretty, 2010; Korpela et al., 2017a). However, most of this past work has focused primarily on physical activities, has not examined outdoor activities at work, and it is unclear whether these benefits translate to work-related outcomes.

One noteworthy exception is Hyvönen et al.’s (2018) person-centered study, which revealed that Finnish employees in profiles characterized by the most frequent visits to natural environments during work and leisure time experienced greater engagement at work than those in profiles characterized by the least frequent visits (Hyvönen et al., 2018). Across all profiles in their study, most participants reported “enjoying nature and natural scenery,” “relaxing and dwelling,” and “walking and jogging,” as leisure outdoor activities (Hyvönen et al., 2018). Due to this, profiles were characterized by the frequency of time spent in natural settings and *variability* of activities engaged in, rather than the specific type of activities. In work-specific studies, researchers have explored how *general* activities (i.e., not outdoor-specific) relate to energy replenishment. Trougakos et al. (2014) found that relaxing lunch breaks reduced fatigue at the end of the workday, whereas social and work-related breaks increased fatigue, particularly for employees with low autonomy. In Bennett et al.’s (2020) study, employees who engaged in microbreaks at work that allowed for mental disengagement from one’s work (i.e., detachment) reported reduced fatigue. Other work has found that spending time on leisure activities during nonwork time (e.g., social, low effort, or physical activities), but not work or household related activities, predicts greater engagement at work (ten Brummelhuis and Bakker, 2012). Collectively, previous research has not explored outdoor activities, both at work and home, alongside work-specific outcomes. It is therefore unknown whether specific activities are associated with engagement and creativity at work.

*Research Question 2:* Are the activities in which individuals engage when they are outside associated with engagement and creativity at work?

## 2 Methods

### 2.1 Participants and procedure

**Figure 2:**
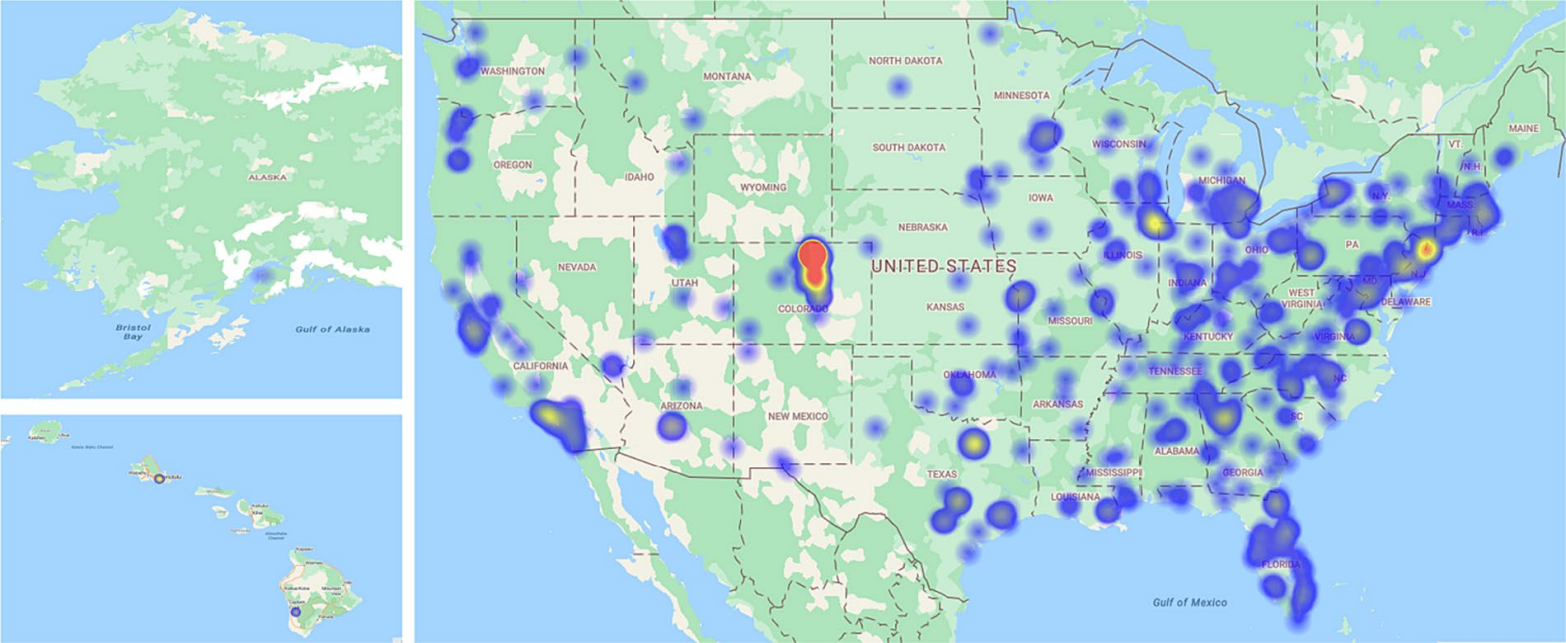
Heat map of participant locations. Heat map depicts the home locations of participants in the present study.

We collected three sets of data for this study. An employee sample was collected from Amazon’s Mechanical Turk (MTurk; December 2019–January 2020, *N* = 564), and two samples were collected from employed undergraduate students (*N* = 239), one in the fall semester (December 2019; *N* = 90), and one in the following spring semester (March 2020–May 2020; *N* = 149). Participants recruited from MTurk lived and worked in different geographic regions across the United States, whereas working student participants were recruited from a large state university in northern Colorado (see Figure 2 for heat map of participant locations). All participants completed the same 15-min online survey and responses from all three data collections were combined for analyses (*N* = 803). The samples were combined because (a) all students were enrolled at the same university, so the built and natural environment where students work and live was homogeneous, thus limiting the variance in urbanity and natural amenities, (b) combining samples produces a larger sample size and greater power to detect indirect effects (Fritz and MacKinnon, 2007), and (c) to account for potential season (i.e., winter versus spring) and COVID-related effects. As explained below, demographic characteristics that vary between samples were modeled as control variables, thus accounting statistically for any differences in demographic characteristics between samples (e.g., age, work hours). Although we combined the samples, we also conducted analyses among the separate samples to investigate whether the decision to combine samples affected the results.

### 2.2 Measures

#### 2.2.1 Built environment

To assess the built environment, the urban–rural classification scheme, developed by the National Center for Health Statistics, was used to assess *urbanity* (Ingram and Franco, 2014). State and county information is required to link participants with urban–rural categories, so participants were asked to report the state, county, and zip code of their work and home addresses. In the development of the urban–rural classification scheme, each county in the United States was categorized broadly based on population size, drawing from 2010 U.S. Census data in conjunction with the US Government Office of Management and Budget standards for defining core-based statistical areas. There are six categories in the urban–rural classification scheme, with four metropolitan (metro) categories (i.e., large central metro, large fringe metro, medium metro, small metro), and two nonmetropolitan categories (i.e., micropolitan, and non-core). The *large central metro* category (e.g., Los Angeles County, California; New York county, New York) represents counties in metropolitical statistical areas (MSAs) with at least one million people and either contain the entire population of the largest city in the MSA, have their entire population within the largest city of the MSA, or contain at least 250,000 residents of any large city in the MSA. The *large fringe metro* category also includes counties in MSAs with at least one million people, but do not otherwise qualify as large central metro counties (e.g., Essex County, Massachusetts). The *Medium metro* category includes counties in MSAs with populations of 250,000–999,999 (e.g., Larimer County, Colorado). The *Small metro* category includes counties in MSAs with populations less than 250,000. The *Micropolitan* category includes counties in micropolitan statistical areas with a population less than 50,000 but include an urban core of at least 10,000 people. Finally, the *non-core* category includes counties that do not contain an urban core with a population of at least 10,000, and therefore have the smallest population sizes and are the most rural (e.g., Big Horn County, Wyoming). In this study, higher scores reflect more urban areas, and lower scores reflect more rural areas.

#### 2.2.2 Natural environment

To assess the natural environment, a scale developed by the US Department of Agriculture was used to assess *natural amenities* (McGranahan, 1999). Like the urban–rural classification system, scores are identified by linking participants’ work and home addresses with the natural amenities scale. Areas with greater natural amenities are characterized by having warm and sunny winters, mild temperatures and low humidity in the summer, access to water, and being hilly or mountainous. Scores are derived from archival data based on average hours of sunlight in January, average temperatures in January and July, average relative humidity in July, proportion of water area (e.g., lakes, ocean), and topographic information based on land surface topography (McGranahan, 1999). The natural amenities scale is on a seven-point scale, with higher scores reflecting areas with greater natural amenities (e.g., Napa County, California) and lower scores reflecting areas with fewer natural amenities (e.g., Champaign County, Illinois).

#### 2.2.3 Outdoor experiences

##### 2.2.3.1 Time outside

Participants were asked to report the total amount of time, in hours, they spent outside in the last week across three settings (i.e., at work, on days off, and before and after work). Specifically, the following questions were asked: “How many hours did you spend outside during work time last week?,” “How many hours did you spend outside during your days off (e.g., the weekend) last week?,” and “How many hours did you spend outside during your nonwork time (i.e., before and after work) last week?.” Responses to the questions about days off and before and after work were summed to reflect the nonwork domain.

##### 2.2.3.2 Enjoyment of time outside

Participants were asked about the extent to which they enjoyed the time they spent outside last week. These questions were presented to participants immediately after they were asked about the amount of time they spent outside. Items were adapted from the lunch break enjoyment item used in Sianoja et al. (2018) paper. Single-item measures were used to assess enjoyment of time spent outside at work (“I enjoyed the time I spent outside during work last week”), and during nonwork time (“I enjoyed the time I spent outside during my days off [e.g., the weekend] last week”) and (“I enjoyed the time I spent outside during my nonwork time [i.e., before and after work] last week”). Response options range from 1 (*strongly disagree*) to 5 (*strongly agree*), with higher scores indicating greater enjoyment of time spent outside in nature. Scores for the responses pertaining to days off and before and after work were averaged to reflect the nonwork domain.

##### 2.2.3.3 Outdoor activities

In the same online surveys, participants were asked to describe qualitatively how they spent their time outside during work and nonwork time in the last week for each setting (i.e., at work, on days off, before and after work). Responses about how time was spent on days off and before and after work were combined for the present study, given the focus on comparisons between work and nonwork time. There was no limit to how much text participants could add, though most responses ranged from one word to a few sentences in length. As described below, responses were qualitatively coded to generate outdoor activity categories.

#### 2.2.4 Job engagement

Rich et al.’s (2010) 18-item job engagement scale was used to assess the exertion of emotional, cognitive, and physical energies in one’s work role. Separate mean scale scores were created for emotional engagement (example scale item: “I feel energetic about my job”; α = 0.94), cognitive engagement (example scale item: “At work, I concentrate on my job”; α = 0.94), and physical engagement (example scale item: “I exert my full effort to my job”; α = 0.87) and used as separate outcomes in all analyses. Response options were on a one-to-five scale (1 = *Strongly Disagree*; 5 = *Strongly Agree*), with higher scores reflecting greater job engagement.

#### 2.2.5 Creativity at work

A 13-item scale was used to assess creativity at work (Zhou and George, 2001; α = 0.95). In the original scale, items were phrased so supervisors could rate their employees’ creativity and were adapted for the present study to reflect participants’ self-reported creativity at work (example scale items: “I come up with creative solutions to problems”; “I often have new and innovative ideas”). Response options were on a one-to-five scale (1 = *Strongly Disagree*; 5 = *Strongly Agree*), with higher mean scores reflecting greater creativity at work.

#### 2.2.6 Control variables

Variables that relate to substantive constructs in the hypothesized models were selected as controls to account for alternative or spurious explanations of findings (Spector and Brannick, 2011). Accordingly, gender, age, race, number of hours worked in the previous week, work schedule, primary location of work (i.e., indoors or outdoors), and sample were modeled as control variables.

### 2.3 Data cleaning and preliminary screening

First, participants who failed either of the two attention checks included in the survey (e.g., “select strongly disagree”) were excluded. Participants who reported working less than 10 h last week were also excluded, given our interest in work-related outcomes. Next, missing data were explored and addressed following Newman’s (2014) procedures. Univariate outliers were identified and removed using procedures outlined in Tabachnick and Fidell (2013). We report and interpret the results with outliers excluded, given that they reflect participant error (e.g., careless responding or misunderstanding), or participants who were not representative of the intended sample (e.g., individuals who spend large amounts of time outside are considerably different than American adults’ typical outdoor time use; Klepeis et al., 2001; Kellert et al., 2017).

### 2.4 Analytic approach

First, to assess the internal structure and psychometric properties of the study’s latent variables, confirmatory factor analyses (CFAs) were performed for the job engagement scale and the creativity at work scale. Recommendations from Hu and Bentler (1999) and Yu (2002) were used to assess model fit. Specifically, model fit values near the following cutoffs indicate good model fit: a non-significant χ^2^ statistic (i.e., value of *p* greater than 0.05), CFI greater than or equal to 0.95, TLI greater than or equal to 0.95, RMSEA less than or equal to 0.06, and SRMR less than or equal to 0.08.

Fully saturated mediated regression path models in Mplus Version 8 were used to test the hypothesized indirect effects of urbanity and natural amenities at work and home on job engagement and creativity, via amount and enjoyment of time spent outside in the last week, accounting for controls. Bootstrapping with 5,000 bias-corrected bootstrapped samples was employed, based on Preacher and Hayes (2008) recommendations, and given that this method is the most powerful for tests of mediation (Fritz and MacKinnon, 2007). Asymmetrical confidence intervals were assessed because they more accurately represent the distribution of the product of coefficients, which is not normally distributed (MacKinnon et al., 2004). Therefore, significance of indirect effects was determined by 95% bias-corrected bootstrapped asymmetrical confidence intervals that did not include zero. See Table 1 for sample sizes, descriptive statistics, and correlations among study variables.

**Table 1:** Descriptive statistics and correlations among study variables.

Variable	*N*	*M*	*SD*	1	2	3	4	5	6	7	8	9	10	11	12	13	14	15	16	17	18	19
1. Natural amenities (W)	796	4.44	1.45	-																		
2. Urbanity (W)	800	4.40	1.29	0.05	-																	
3. Natural amenities (H)	797	4.44	1.44	0.97**	0.06	-																
4. Urbanity (H)	801	4.39	1.29	0.05	0.92**	0.06	-															
5. Gender	798	0.59	0.49	0.02	-0.08*	0.02	-0.07*	-														
6. Age	801	32.99	12.77	-0.38**	0.06	-0.38**	0.05	0.00	-													
7. Race	803	0.66	0.48	-0.14**	-0.08*	-0.13**	-0.09*	0.00	0.06	-												
8. Work hours last week	803	33.20	11.43	-0.37**	0.06	-0.37**	0.05	-0.16**	0.40**	-0.01	-											
9. Work schedule	803	0.54	0.50	-0.27**	0.05	-0.26**	0.04	-0.05	0.29**	0.05	0.40**	-										
10. Work location	802	0.96	0.20	-0.10**	0.02	-0.10**	0.05	0.06	0.13**	-0.05	0.06	0.02	-									
11. Student fall sample	803	0.11	0.32	0.35**	-0.07*	0.36**	-0.07*	0.05	-0.38**	-0.01	-0.38**	-0.30**	-0.04	-								
12. Student spring sample	803	0.19	0.39	0.42**	-0.08*	0.41**	-0.07	0.03	-0.48**	-0.07	-0.39**	-0.26**	-0.20**	-0.17**	-							
13 Time outside (W)	698	2.59	3.91	0.13**	0.07	0.12**	0.04	-0.06	-0.03	-0.00	0.04	-0.05	-0.43**	0.04	0.06	-						
14. Time outside (NW)	792	9.83	8.45	0.28**	0.04	0.29**	0.04	-0.05	-0.15**	-0.03	-0.12**	-0.14**	-0.05	0.11**	0.23**	0.25**	-					
15. Enjoy outside (W)	476	4.01	0.78	-0.01	0.01	-0.00	-0.02	0.04	0.07	-0.06	0.03	0.08	-0.05	-0.04	-0.02	0.14**	0.03	-				
16. Enjoy outside (NW)	555	4.18	0.67	0.12**	-0.06	0.14**	-0.07	0.01	-0.05	-0.07	-0.07	-0.05	0.00	-0.01	0.13**	0.03	0.28**	0.35**	-			
17. Emotional engagement	803	3.63	0.90	-0.02	0.02	-0.04	0.03	0.03	0.13**	-0.02	0.06	0.05	0.03	-0.11**	-0.02	0.06	0.09**	0.22**	0.18**	-		
18. Cognitive engagement	802	3.91	0.76	-0.10**	-0.01	-0.10**	-0.01	0.07	0.26**	-0.04	0.16**	0.07*	0.05	-0.15**	-0.11**	-0.03	-0.03	0.18**	0.08	0.58**	-	
19. Physical engagement	803	3.92	0.64	-0.03	-0.06	-0.04	-0.05	0.11**	0.14**	-0.04	0.10**	0.00	0.00	-0.08*	-0.03	0.03	0.03	0.16**	0.11*	0.49**	0.70**	-
20. Creativity	802	3.46	0.78	-0.07	0.02	-0.06	0.01	-0.01	0.16**	-0.04	0.18**	0.11**	0.03	-0.09*	-0.09**	0.06	0.04	0.21**	0.14**	0.51**	0.44**	0.46**

W = Work, H = Home, NW = Nonwork. Gender (0 = Male, 1 = Female). Race (0 = People of Color, 1 = White). Work Schedule (0 = Other, 1 = Regular Daytime Schedule). Work Location (0 = Primarily Outdoors, 1 = Primarily Indoors). Student Fall Sample (1 = Participant in Student Fall 2019 Sample, 0 = Participant in Student Spring 2020 Sample or MTurk Sample), Student Spring Sample (1 = Participant in Student Spring 2020 Sample, 0 = Participant in Student Fall 2019 Sample or MTurk Sample). *p < 0.05, **p < 0.01.

For the qualitative responses, content analysis was used to identify outdoor activity categories (Schreier, 2012). Content analysis is particularly useful when analyzing large amounts of text-based data and when the unit of analysis is a simple word or phrase (e.g., Krippendorff, 2004; Braun and Clarke, 2006). In line with best practices for qualitative coding, a series of precoding, coding, and recoding activities were performed in a collaborative process conducted by two independent coders (e.g., Ritchie et al., 2013; Saldaña, 2016; Ravitch and Carl, 2019). Our detailed codebook included mutually exclusive categories with descriptive names, definitions, inclusion and exclusion criteria, typical examples, and “close, but no” examples (Bernard and Ryan, 2010; Schreier, 2012). The final round of coding was conducted for all 803 participants, with 24,090 coding decisions, resulting in 98% overall agreement and an overall kappa value of 0.92, which is considered a nearly perfect level of agreement (McHugh, 2012). Associations between outdoor activities and work outcomes, with all control variables included, were explored using linear regression analyses in SPSS Version 26.

## 3 Results

### 3.1 Confirmatory factor analyses

A single-factor CFA for the job engagement scale had poor model fit; χ^2^(135) = 4101.42, *p* < 0.05, CFI = 0.67, TLI = 0.63, RMSEA = 0.19, SRMR = 0.11. In line with the theoretical conceptualization of job engagement as having dimensions for emotional, cognitive, and physical engagement at work, a three-factor model was tested next. The three-factor CFA had adequate model fit; χ^2^(132) = 1017.14, *p* < 0.05, CFI = 0.93, TLI = 0.92, RMSEA = 0.09, SRMR = 0.04, and demonstrated significant improvement in model fit compared to the single-factor model (Δ χ^2^ = 3084.28, *p* < 0.001). Therefore, three separate dimensions of job engagement (emotional, cognitive, and physical) are examined across analyses. The single-factor CFA for the creativity at work measure had sufficient model fit; χ^2^(65) = 515.94, *p* < 0.05, CFI = 0.94, TLI = 0.93, RMSEA = 0.09, SRMR = 0.03.

### 3.2 Indirect effects^1^^,^^2^

Tables 2, 3 depict the direct effects among the built and natural environment (i.e., urbanity and natural amenities), mediators (i.e., time outside and enjoyment of time outside, respectively), and work-related outcomes. Table 4 provides the 95% confidence intervals (CIs) for the indirect effects; CIs that do not include zero (bolded in the table) reflect significant indirect effects.

**Table 2:** Hours outside as a mediator: direct effects among the built and natural environment, hours spent outside, and work-related outcomes.

	Mediator	Outcomes			
	Time outside (W)	Emotional engagement	Cognitive engagement	Physical engagement	Creativity
Predictor	*b* (*SE*)	*b* (*SE*)	*b* (*SE*)	*b* (*SE*)	*b* (*SE*)
Constant	7.85 (1.80)	3.00 (0.33)	3.31 (0.25)	3.61 (0.22)	2.65 (0.28)
Student fall sample	−0.02 (0.71)	−0.25 (0.15)	−0.10 (0.12)	−0.05 (0.10)	0.08 (0.13)
Student spring sample	0.07 (0.63)	0.02 (0.12)	0.02 (0.10)	0.03 (0.09)	0.05 (0.11)
Work hours last week	0.05 (0.02)	−0.00 (0.004)	0.01 (0.003)	0.01 (0.003)	0.01 (0.003)
Work schedule	−0.36 (0.33)**	0.02 (0.07)	−0.04 (0.06)	−0.07 (0.05)	0.09 (0.06)
Work location	−9.34 (1.44)***	0.30 (0.20)	0.02 (0.16)	−0.03 (0.14)	0.21 (0.18)
Gender	−0.18 (0.28)	0.08 (0.07)	0.12 (0.06)	0.16 (0.05)**	0.03 (0.06)
Age	0.00 (0.02)	0.01 (0.003)*	0.01 (0.003)***	0.01 (0.002)**	0.01 (0.003)**
Race	−0.04 (0.31)	−0.05 (0.07)	−0.08 (0.06)	−0.07 (0.05)	−0.08 (0.06)
Natural amenities (W)	0.35 (0.15)*	0.02 (0.03)	0.01 (0.02)	0.01 (0.02)	0.00 (0.03)
Urbanity (W)	0.16 (0.11)	0.002 (0.03)	−0.01 (0.02)	−0.03 (0.02)†	0.00 (0.02)
Time outside (W)		0.02 (0.01)*	−0.004 (0.01)	0.004 (0.01)	0.02 (0.01)†
Model *R*^2^	0.26	0.03	0.08	0.05	0.05
Constant	0.36 (2.81)	3.19 (0.31)	3.27 (0.25)	3.63 (0.21)	2.78 (0.28)
Student fall sample	2.78 (1.62)†	−0.26 (0.16)†	−0.11 (0.12)	−0.05 (0.10)	0.04 (0.13)
Student spring sample	4.66 (1.32)***	−0.02 (0.12)	0.03 (0.10)	0.04 (0.09)	0.00 (0.11)
Work hours last week	0.04 (0.04)	0.00 (0.004)	0.01 (0.003)†	0.01 (0.003)†	0.01 (0.003)**
Work schedule	−0.71 (0.66)	0.02 (0.07)	−0.03 (0.06)	−0.07 (0.05)	0.09 (0.06)
Work location	0.16 (1.55)	0.08 (0.17)	0.06 (0.15)	−0.06 (0.12)	0.05 (0.15)
Gender	−0.94 (0.60)	0.08 (0.07)	0.13 (0.06)*	0.16 (0.05)**	0.03 (0.06)
Age	0.04 (0.03)	0.01 (0.003)*	0.01 (0.003)***	0.01 (0.002)**	0.01 (0.003)**
Race	0.24 (0.62)	−0.06 (0.07)	−0.08 (0.06)	−0.07 (0.05)	−0.08 (0.06)
Natural amenities (H)	1.18 (0.27)***	−0.01 (0.03)	0.01 (0.02)	0.00 (0.02)	0.01 (0.03)
Urbanity (H)	0.24 (0.20)	0.01 (0.03)	−0.02 (0.02)	−0.03 (0.02)	−0.01 (0.02)
Time outside (NW)		0.01 (0.004)***	0.00 (0.003)	0.00 (0.003)	0.01 (0.002)*
Model *R*^2^	0.11	0.04	0.08	0.05	0.05

See Table 1 for variable coding information. †*p* < 0.10, *p < 0.05, **p < 0.01, ***p < 0.001.

**Table 3:** Enjoyment outside as a mediator: direct effects among the built and natural environment, enjoyment of time spent outside, and work-related outcomes.

	Mediator	Outcomes			
	Enjoy outside (W)	Emotional engagement	Cognitive engagement	Physical engagement	Creativity
Predictor	*b* (*SE*)	*b* (*SE*)	*b* (*SE*)	*b* (*SE*)	*b* (*SE*)
Constant	4.16 (0.35)	2.11 (0.40)	2.66 (0.29)	3.17 (0.27)	1.98 (0.33)
Student fall sample	−0.04 (0.16)	−0.24 (0.15)	−0.10 (0.12)	−0.05 (0.10)	0.09 (0.13)
Student spring sample	−0.01 (0.14)	0.02 (0.12)	0.03 (0.10)	0.04 (0.09)	0.06 (0.11)
Work hours last week	−0.00 (0.01)	0.00 (0.004)	0.01 (0.003)*	0.01 (0.003)*	0.01 (0.003)**
Work schedule	0.11 (0.08)	−0.02 (0.07)	−0.05 (0.06)	−0.08 (0.05)	0.07 (0.06)
Work location	−0.23 (0.13)†	0.14 (0.18)	0.09 (0.15)	−0.04 (0.12)	0.10 (0.15)
Gender	0.08 (0.07)	0.05 (0.07)	0.11 (0.06)*	0.15 (0.05)**	0.01 (0.06)
Age	0.01 (0.003)	0.01 (0.003)†	0.01 (0.003)***	0.01 (0.002)**	0.01 (0.003)*
Race	−0.11 (0.08)	−0.02 (0.07)	−0.07 (0.06)	−0.05 (0.05)	−0.06 (0.06)
Natural Amenities (W)	0.00 (0.03)	0.02 (0.03)	0.01 (0.02)	0.02 (0.02)	0.01 (0.03)
Urbanity (W)	−0.01 (0.03)	0.01 (0.03)	−0.01 (0.02)	−0.03 (0.02)†	0.01 (0.02)
Enjoy Outside (W)		0.26 (0.06)***	0.15 (0.05)**	0.12 (0.04)**	0.19 (0.05)***
Model *R*^2^	0.02	0.07	0.10	0.07	0.08
Constant	3.96 (0.28)	2.11 (0.42)	2.82 (0.33)	3.18 (0.28)	1.94 (0.35)
Student fall sample	−0.09 (0.12)	−0.19 (0.16)	−0.09 (0.12)	−0.02 (0.10)	0.08 (0.13)
Student spring sample	0.13 (0.10)	0.01 (0.12)	0.02 (0.10)	0.04 (0.09)	0.01 (0.11)
Work hours last week	−0.00 (0.004)	0.00 (0.004)	0.01 (0.003)†	0.01 (0.003)†	0.01 (0.003)**
Work schedule	0.01 (0.07)	0.01 (0.07)	−0.04 (0.06)	−0.07 (0.05)	0.09 (0.06)
Work location	0.10 (0.16)	0.06 (0.18)	0.05 (0.15)	−0.07 (0.12)	0.03 (0.15)
Gender	−0.01 (0.06)	0.07 (0.07)	0.13 (0.05)*	0.16 (0.05)**	0.03 (0.06)
Age	0.00 (0.003)	0.01 (0.003)*	0.01 (0.003)***	0.01 (0.002)**	0.01 (0.003)*
Race	−0.06 (0.06)	−0.04 (0.07)	−0.08 (0.06)	−0.06 (0.05)	−0.07 (0.06)
Natural amenities (H)	0.07 (0.03)**	−0.01 (0.03)	−0.00 (0.02)	0.00 (0.02)	0.00 (0.03)
Urbanity (H)	−0.04 (0.02)	0.02 (0.03)	−0.01 (0.02)	−0.02 (0.02)	0.01 (0.02)
Enjoy outside (NW)		0.28 (0.07)***	0.12 (0.06)*	0.11 (0.05)*	0.21 (0.06)***
Model *R*^2^	0.04	0.07	0.09	0.06	0.08

See Table 1 for variable coding information. †*p* < 0.10, *p < 0.05, **p < 0.01, ***p < 0.001.

**Table 4:** Indirect effects.

Indirect effect	*b*	*SE*	95% CI (upper, lower)
Urbanity (W) ➔Hours (W) ➔Emo. Engage	0.004	0.003	0.000, 0.011
Urbanity(W) ➔Hours (W) ➔Cog. Engage	−0.001	0.001	−0.006, 0.001
Urbanity (W) ➔ Hours (W) ➔Phys. Engage	0.001	0.001	−0.001, 0.005
Urbanity (W) ➔Hours (W) ➔Creativity	0.003	0.002	0.000, 0.010
Natural Amenities (W) ➔Hours (W) ➔Emo. Engage	0.008	0.005	**0.001, 0.020**
Natural Amenities (W) ➔ Hours (W) ➔Cog. Engage	−0.001	0.003	−0.009, 0.003
Natural Amenities (W) ➔Hours (W) ➔Phys. Engage	0.001	0.002	−0.002, 0.008
Natural Amenities (W) ➔Hours (W) ➔Creativity	0.006	0.004	0.000, 0.016
Urbanity (H) ➔Hours (NW) ➔Emo. Engage	0.003	0.003	−0.001, 0.010
Urbanity(H) ➔Hours (NW) ➔Cog. Engage	0.000	0.001	−0.001, 0.004
Urbanity (H) ➔ Hours (NW) ➔ Phys. Engage	0.001	0.001	0.000, 0.005
Urbanity (H) ➔Hours (NW) ➔Creativity	0.002	0.002	−0.001, 0.007
Natural Amenities (H) ➔Hours (NW) ➔Emo. Engage	0.016	0.006	**0.007, 0.030**
Natural Amenities (H) ➔Hours (NW) ➔Cog. Engage	0.002	0.004	−0.006, 0.010
Natural Amenities (H) ➔Hours (NW) ➔Phys. Engage	0.005	0.004	−0.003, 0.013
Natural Amenities (H) ➔Hours (NW) ➔Creativity	0.009	0.005	**0.001, 0.020**
Urbanity (W) ➔Enjoy (W) ➔Emo. Engage	−0.003	0.008	−0.019, 0.012
Urbanity(W) ➔Enjoy (W) ➔Cog. Engage	−0.002	0.005	−0.012, 0.007
Urbanity (W) ➔Enjoy (W) ➔Phys. Engage	−0.001	0.004	−0.010, 0.005
Urbanity (W) ➔Enjoy (W) ➔Creativity	−0.002	0.006	−0.014, 0.010
Natural Amenities (W) ➔Enjoy (W) ➔Emo. Engage	0.001	0.008	−0.015, 0.016
Natural Amenities (W) ➔Enjoy (W) ➔Cog. Engage	0.000	0.005	−0.009, 0.009
Natural Amenities (W) ➔Enjoy (W) ➔Phys. Engage	0.000	0.004	−0.008, 0.007
Natural Amenities (W) ➔Enjoy (W) ➔Creativity	0.001	0.006	−0.011, 0.013
Urbanity (H) ➔Enjoy (NW) ➔Emo. Engage	−0.011	0.007	−0.027, 0.001
Urbanity(H) ➔Enjoy (NW) ➔Cog. Engage	−0.004	0.004	−0.016, 0.000
Urbanity (H) ➔Enjoy (NW) ➔Phys. Engage	−0.004	0.004	−0.014, 0.000
Urbanity (H) ➔Enjoy (NW) ➔Creativity	−0.008	0.006	−0.021, 0.001
Natural Amenities (H) ➔Enjoy (NW) ➔Emo. Engage	0.018	0.008	**0.005, 0.038**
Natural Amenities (H) ➔Enjoy (NW) ➔Cog. Engage	0.008	0.005	**0.001, 0.021**
Natural Amenities (H) ➔Enjoy (NW) ➔Phys. Engage	0.007	0.004	**0.001, 0.019**
Natural Amenities (H) ➔Enjoy (NW) ➔Creativity	0.014	0.007	**0.004, 0.030**

All values were obtained from 5,000 bias-corrected bootstrapped samples in fully saturated path models testing direct and indirect effects. b = Unstandardized Indirect Effect. SE = Standard Error. CI = Confidence Interval. W = Work. H = Home. NW = Nonwork. Emo. Engage = Emotional Engagement. Cog. Engage = Cognitive Engagement. Phys. Engage = Physical Engagement. Hours = Hours Spent Outside. Enjoy = Enjoyment of Time Spent Outside. Bolded 95% CIs indicate significant indirect effects.

#### 3.2.1 Built environment

There were no significant indirect effects of urbanity at work or home on job engagement or creativity outcomes via time spent outside or enjoyment of time outside.

#### 3.2.2 Natural environment

Regarding *time spent outside*, after controlling for all other variables in the model, there was a significant indirect effect of natural amenities at work on emotional job engagement through hours spent outside at work (indirect effect = 0.008, 95% CI [0.001, 0.020]). Similarly, there was an indirect effect of natural amenities at home on emotional job engagement through nonwork hours spent outside (indirect effect = 0.016, 95% CI [0.007, 0.030]). Additionally, there was an indirect effect of natural amenities at home on creativity through nonwork hours spent outside (indirect effect = 0.009, 95% CI [0.001, 0.020]). Regarding *enjoyment of time spent outside*, after controlling for all other variables in the model, there were significant indirect effects of natural amenities at home on physical job engagement (indirect effect = 0.007, 95% CI [0.001, 0.019]), emotional job engagement (indirect effect = 0.018, 95% CI [0.005, 0.038]), cognitive job engagement (indirect effect = 0.008, 95% CI [0.001, 0.021]), and creativity (indirect effect = 0.014, 95% CI [0.004, 0.030]), through enjoyment of nonwork time outside.

### 3.3 Qualitative results

**Figure 3:**
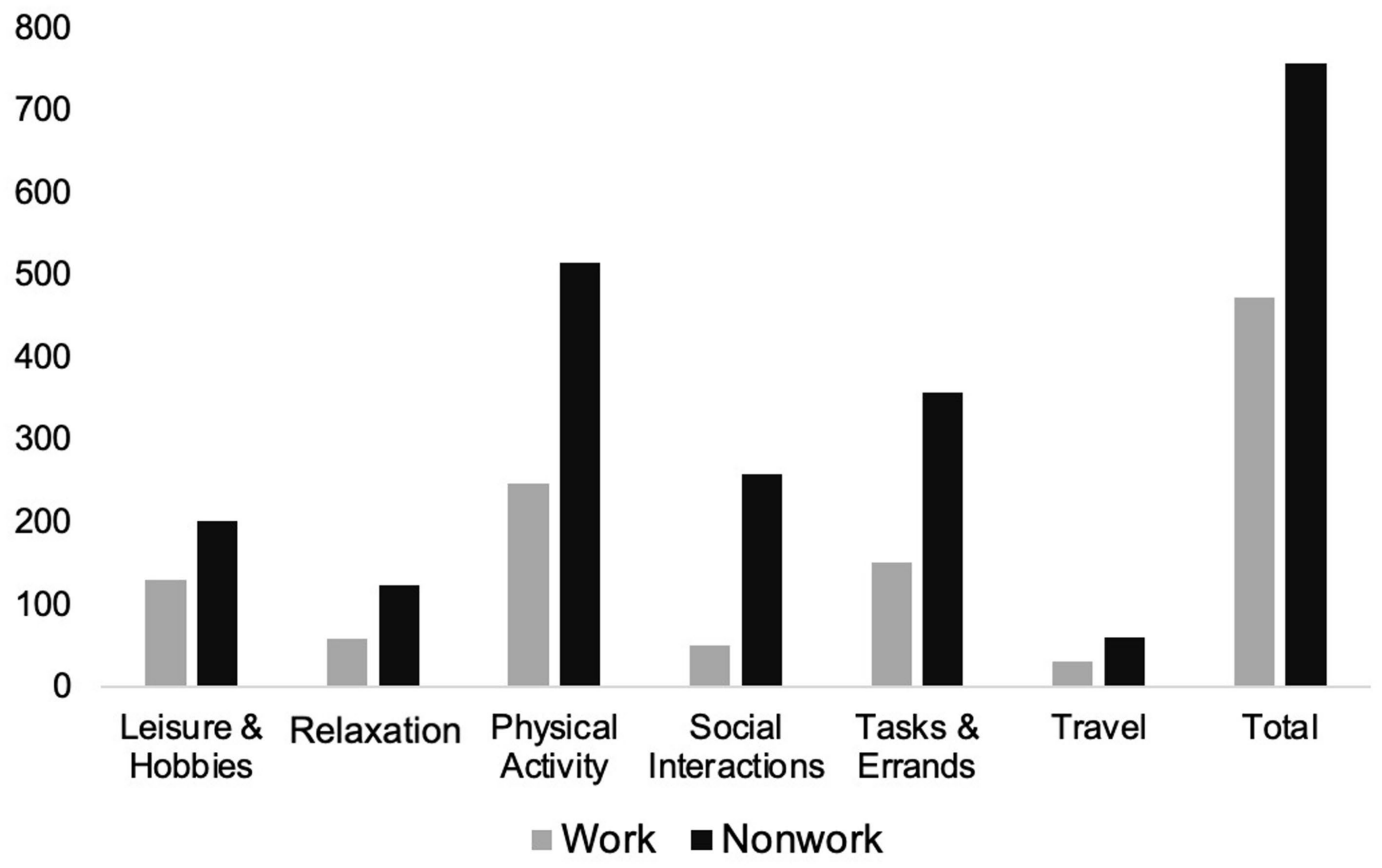
Outdoor activities. Figure reflects the number of participants who reported engaging in different outdoor activities in the last week.

**Figure 4:**
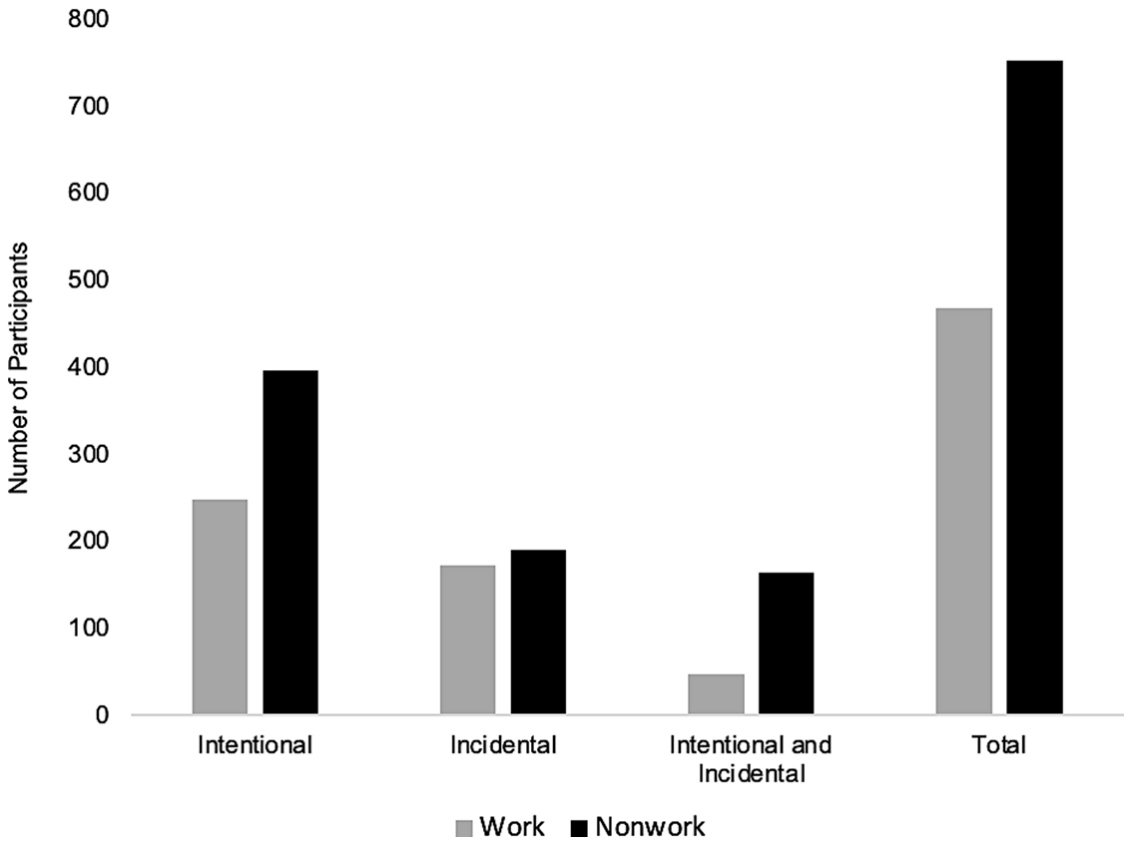
Intentional and incidental outdoor activities. Figure reflects the number of participants who reported engaging in different outdoor activities in the last week.

Although findings may be reported entirely qualitatively (e.g., describing themes and categories), quantifying qualitative data is common in content analysis and can be effective for studies with large sample sizes (Krippendorff, 2004; Braun and Clarke, 2006; Schreier, 2012; Pluye and Hong, 2014). Therefore, we report descriptive quantitative findings gleaned from the qualitative responses (see Figures 3, 4), and test associations between outdoor activities and work-related outcomes.

#### 3.3.1 Outdoor activities at work and home

There were six primary ways that participants spent their time outside: leisure activities and hobbies (e.g., reading), relaxation (e.g., relaxing or reflecting), physical activities (e.g., playing sports), social interactions (e.g., spending time with family), tasks and errands (e.g., yardwork, shopping), and traveling (e.g., driving). Overall, participants spent more nonwork time outside (*M* = 9.83 h during the last week) compared to during work time (*M* = 2.59 h during the last week). It was more common for participants to report not spending any time outside during work time compared to nonwork time (see Figure 3). Most participants reported only one outdoor activity at work (range of 1–4 activities), compared to during nonwork time, which was spent on more varied activities (range of 1–5, with approximately one-third of participants reporting three or more activities). Regardless of setting, outdoor physical activities were reported the most frequently, followed by tasks and errands. However, a greater proportion of participants performed physical activities and tasks and errands outside during nonwork time compared to work time. Workers engaged in leisure activities and hobbies to a similar extent (proportionately) during work and nonwork time. Of note is that outdoor social and relaxation activities were more common during nonwork time than work time. Traveling was mentioned the least frequently and at comparable proportions across work and nonwork settings (see Figure 3).

#### 3.3.2 Intentionality of outdoor activities

During the qualitative coding process, we noticed that some participants explicitly indicated that they were only outside as a byproduct of another task, particularly for traveling to places and running errands, whereas others would describe intentionally spending time outside. Therefore, an additional coding process, drawing from Keniger et al. (2013) distinction between incidental and intentional experiences of nature, was performed. Responses were categorized as being intentional (i.e., purposefully being outside), incidental (i.e., being outside as a by-product of another activity), or both.^3^

Illustratively, the following participant response describes going outside *incidentally*: “I was outside as a byproduct of trying to get from one place to another. I was not paying much attention to my surroundings and I was not outside for very long.” It was common for participants to report simply walking to and from their car, or driving to and from destinations (e.g., “Running errands – getting in and out of the car, and walking to and from and between buildings”; “We also went outside to run errands, though the act of being outside was merely to drive to the destination.”). In contrast, the following responses describe going outside *intentionally*: “I need time to think and be away from the demands of the job. Just seeing the green grass and being alone helps to clear my mind for the rest of the day. It has become a ritual to me. I go, I sit, I think.” and: “I also spent some time on the nature trail as well. I was really stressed out one day and needed to be around the quiet calm of nature. I feel like doing some light hiking through nature was very calming for me.” During work and nonwork time, more participants reported intentional outdoor activities, followed by incidental outdoor activities, with a combination of both intentional and incidental outdoor activities being the least frequent, particularly during work time (see Figure 4).

#### 3.3.3 Associations between outdoor activities and work outcomes

##### 3.3.3.1 Outdoor activities during work

Using linear regression analyses with all control variables accounted for, there was a significant and positive association between activities involving tasks and errands during work time and cognitive engagement (*B* = 0.20, *SE* = 0.08, *p* = 0.01, *R*^2^ = 0.07). No other outdoor activities during work time were significantly associated with emotional engagement, cognitive engagement, physical engagement, or creativity.

##### 3.3.3.2 Outdoor activities during nonwork

Nonwork outdoor physical activities (*B* = 0.21, *SE* = 0.07, *p* < 0.01, *R*^2^ = 0.03) and social interactions (*B* = 0.16, *SE* = 0.07, *p* = 0.03, *R*^2^ = 0.03) were significantly and positively associated with emotional engagement, and nonwork outdoor social activities (*B* = 0.13, *SE* = 0.05, *p* < 0.01, *R*^2^ = 0.06) and tasks and errands (*B* = 0.13, *SE* = 0.05, *p* < 0.01, *R*^2^ = 0.06) were significantly and positively associated with physical engagement. No other outdoor activities during nonwork time were significantly associated with emotional engagement, cognitive engagement, physical engagement, or creativity.

### 3.4 Supplemental exploratory analyses

To explore whether different types of outdoor activities were associated with the environment where participants live and work, or their experience of the environment, supplemental point-biserial correlational analyses were performed (Tabachnick and Fidell, 2013) and presented in Table 5.

**Table 5:** Point-biserial correlations among study variables and outdoor activities.

	Leisure & hobbies	Relaxation	Physical activity	Social	Tasks & errands	Traveling	Intentional	Incidental	Intentional & incidental
Work									
Natural amenities (W)	0.00	−0.11*	−0.01	−0.02	0.06	0.02	−0.04	0.02	0.02
Urbanity (W)	0.06	−0.05	0.05	0.08	−0.04	0.08	0.06	−0.09	0.05
Time outside (W)	−0.01	−0.01	0.00	−0.08	0.12**	0.15**	−0.06	−0.01	0.11*
Enjoy outside (W)	0.11*	0.09*	0.02	0.08	−0.06	−0.06	0.19**	−0.21**	0.01
Emotional engagement	0.02	0.02	0.01	0.00	0.05	0.03	−0.04	0.03	0.02
Cognitive engagement	0.09	0.05	−0.04	0.01	0.11*	−0.03	−0.04	0.02	0.04
Physical engagement	0.05	0.05	−0.10*	0.08	0.11*	−0.03	−0.07	0.05	0.03
Creativity	0.04	0.01	−0.01	0.01	0.04	0.05	−0.01	−0.01	0.03
Nonwork									
Natural amenities (H)	0.17**	0.10**	0.18**	−0.04	−0.06	−0.05	0.19**	−0.20**	−0.03
Urbanity (H)	0.05	−0.02	0.05	0.01	−0.01	0.08*	−0.01	−0.01	0.02
Time outside (NW)	0.14**	0.11**	0.22**	0.07	0.02	−0.02	0.14**	−0.20**	0.05
Enjoy outside (NW)	0.12**	0.11**	0.18**	0.12**	−0.04	−0.09*	0.22**	−0.29**	0.01
Emotional engagement	−0.03	−0.03	0.08*	0.09*	0.01	−0.06	0.04	−0.06	0.02
Cognitive engagement	−0.02	−0.03	0.01	0.07*	0.09*	−0.04	−0.07	0.05	0.04
Physical engagement	0.01	−0.01	−0.01	0.12**	0.12**	−0.07*	−0.05	0.01	0.06
Creativity	0.01	−0.04	−0.03	0.06	0.03	−0.04	0.03	−0.03	0.00

W, Work. H, Home. NW, Nonwork. Note that control variables are not accounted for in these simple point-biserial correlations. *p < 0.05, **p < 0.01.

#### 3.4.1 Natural amenities and urbanity

Regarding the surrounding environment where participants live, natural amenities at home were positively correlated with outdoor leisure activities and hobbies, relaxation activities, physical activities, and intentional activities, and negatively correlated with incidental activities. There was a positive correlation between living in more urban areas and spending time outside for traveling or transportation. A negative correlation was observed between natural amenities at work and relaxation activities. No other significant correlations were detected among outdoor activities, natural amenities, or urbanity at work and home.

#### 3.4.2 Time spent outside

The activities that were positively correlated with time spent outside differed depending on the setting. At work, tasks and errands, traveling, and a combination of intentional and incidental outdoor activities were correlated with more time spent outside. On the other hand, during nonwork time, leisure activities and hobbies, relaxation, physical activities, and intentional activities were positively correlated with time spent outside, whereas incidental activities were negatively correlated with time spent outside.

#### 3.4.3 Enjoyment of time spent outside

The patterns of correlations between type of activities and enjoyment of time outside were more consistent across settings. During both work and nonwork time, leisure activities and hobbies, relaxation, and intentional activities were positively correlated with enjoyment of time outside, whereas incidental activities were negatively correlated with enjoyment of time spent outside. During nonwork time, physical activities and social activities were also positively correlated with enjoyment of time outside, and being outside for traveling or transportation was negatively correlated with enjoyment.

## 4 Discussion

Results from the present study demonstrate that the benefits of nature extend to motivational work-related outcomes of job engagement and creativity. We provide initial support for tenets of Klotz and Bolino (2021) biophilic work design model and extend the model by evaluating aspects of the surrounding built and natural environments at work and home, and specific work-related outcomes. Specifically, individuals who work in areas with greater natural amenities spend more time outside during the workday, which enables greater feelings of enthusiasm, positivity, and pride about their job (i.e., emotional job engagement). Similarly, individuals who live in areas with more natural amenities also spend more time outside, and experience greater emotional engagement and creativity in their work. Further, individuals who live in areas with greater natural amenities report greater enjoyment of time outside, and in turn report working with high effort and intensity, feeling excited about and interested in their job, having the ability to focus their attention and concentrate on their job, and generating creative and useful ideas for their work. Urbanity was not associated with spending time outside, enjoying time outside, or work-related outcomes. Therefore, we found indirect effects of the natural (but not built) environment at work and home on work-related outcomes.

During nonwork time, outdoor social interactions, physical activities, and tasks and errands were associated with engagement at work. Participants who reported going outside for social interactions during nonwork time reported greater physical and emotional engagement in their job compared to those who did not have outdoor social interactions. This is similar to past research on the widespread benefits of social support, as well as engaging in social activities during leisure time (e.g., Almedom, 2005; Haber et al., 2007; Newman, 2014), but these results are also somewhat discrepant from other work that has found that outdoor social interactions (e.g., being with children) can inhibit restoration (e.g., White et al., 2013). In alignment with self-determination theory, the findings in the present study suggest that social connectivity, or meeting the psychological need of relatedness/affiliation (i.e., feelings of belonging; Ryan and Deci, 2000), may be important for replenishing energies that enable individuals to exert full effort in their work and feel energetic and positive about their job. Next, the positive association between physical activities and emotional engagement aligns with the large literature on the psychological benefits of physical activities (Stathopoulou et al., 2006; Wiese et al., 2018), particularly when exercising outdoors (Barton and Pretty, 2010; Weng and Chiang, 2014). Despite past research that has found that participating in household chores can inhibit job engagement (ten Brummelhuis and Bakker, 2012), participants in this study who performed outdoor tasks and errands also reported being engaged at work, which may be due to greater feelings of accomplishment or competence (Ryan and Deci, 2000). None of the outdoor activity categories were associated with creativity at work. Because researchers have found that employees who are more creative in their nonwork time are also more creative at work (Eschleman et al., 2014), future research could consider qualitatively coding for the degree of creativity in outdoor activities (e.g., wildlife photography compared to eating a meal outside), which may better predict creativity at work. It is possible that the lack of significant findings may be due to the limited amount of time workers in these samples spent outside at work. Future research should re-examine this relationship among workers who may spend more time outside.

Although natural amenities at home and work were highly correlated (*r* = 0.97), they were related to experiences of being outside in different ways, and more effects were found across the nonwork models compared to the work models. This pattern of results highlights the importance of extending the biophilic work design model to the home domain. One possible explanation is that it was common for participants to report not spending any work time outside in the last week. Thus, the fewer effects in the work models could be attributed to the reduced variability in the number of hours individuals spent outside during the workday. In future work, it would be useful to explore boundary conditions of the biophilic work design model in terms of workplace factors that may act as barriers to being able to go outside during the workday, such as a lack of schedule flexibility, job autonomy, availability of work breaks, or workplace norms surrounding the use of work breaks. In addition, recent research indicates that engagement with nature can depend on one’s intent (i.e., pre-planning to engage in certain behaviors) and self-efficacy (i.e., a person’s confidence in their ability to act despite obstacles or challenges; Maddock et al., 2022). Although this area of research is relatively new, organizations and supervisors should build in flexibility in work hours and location, if possible, which can grant employees the opportunity to plan for and carry out their intents to engage with nature (Howe et al., 2022). For example, if someone works in a job that has unpredictable day-level demands or more restrictive work hours and is therefore less flexible (such as on-call nurses), it may hinder the employee’s intent to spend time outside by reducing their perceived self-efficacy to pre-plan for an outdoor work break. These factors may be more predictive of whether employees go outside during the workday than the location of their work environment.

Although spending time outside can be beneficial for motivational work-related outcomes, we found that it is particularly important that time spent outside is enjoyable. In line with past research, enjoyment is a critical feature of leisure time and work breaks (e.g., Kuykendall et al., 2015; Sonnentag et al., 2017), and of greater importance for employee outcomes compared to the length of a work break (Hunter and Wu, 2016; Bennett et al., 2020). Thus, the importance of enjoyment for energy restoration applies to time spent specifically in outdoor settings. Regardless of setting, participants who reported engaging in outdoor leisure activities, hobbies, relaxation, and who reported going outside intentionally rather than incidentally, were more likely to enjoy their time outside than those who did not report these types of outdoor activities (see Table 5). During nonwork time, participants who engaged in physical activities and had social interactions outdoors also reported greater enjoyment of time outside, whereas participants who were outside for travel reported lower enjoyment. Therefore, how time is spent outside is an important factor in whether it is enjoyable. Additionally, participants who live in areas with greater natural amenities were more likely to report spending time outside on leisure activities, hobbies, relaxation, physical activities, and on outdoor activities that are intentional rather than incidental. On the contrary, living in more urban areas was positively correlated with spending time outside for traveling or transportation (see Table 5). These results indicate that the surrounding environment also influences how individuals choose to spend their time outside.

### 4.1 Practical implications

There are a number of ways in which the results from our study can inform practical recommendations for organizations, supervisors, and employees who are interested in improving job engagement and creativity at work. Yet, it is important to note that the magnitude of the significant effects detected in the present study were small, suggesting that the following practical recommendations may be best interpreted provisionally.

At the employee-level, our results suggest that individuals may experience greater engagement and creativity at work if they spend more time outside at work and at home and enjoy the time they spend outside. Certain types of outdoor activities may predict greater time spent outside (e.g., exercising during nonwork time), enjoyment of time outside (e.g., relaxing activities), and job engagement (e.g., social activities during nonwork time). These individual-level approaches would be cost-effective for organizations and could be further reinforced by supervisors who can effectively help employees balance the demands of their work and nonwork lives (Hammer et al., 2009) and encourage employees to spend time outside (e.g., by having outdoor meetings).

At the organizational-level, organizations can implement nature-related policies, such as paid days off to spend outside or discounted State and National Park passes. Instituting and enforcing flexible schedule policies can also enable employees to spend time outside and in enjoyable ways; flexible schedules, including longer breaks during work hours (while not reducing paid work time), could allow employees who want to surf in the morning, hike in the afternoon, sunbathe mid-day, or stargaze in the evening to do so. More substantial workplace redesign efforts can include creating rooftop gardens, installing on-site bike racks, picnic tables, and outdoor game spaces, or creating nearby paved walking or biking trails with wheelchair accessibility. It is important that on-site outdoor spaces are close in proximity to the workplace, comfortable, and suitable for different weather conditions (e.g., include fans in hotter climates, ensure different forms of sun protection) (Petersson Troije et al., 2021).

### 4.2 Limitations and future directions

The primary limitations of this study are methodological, which can be addressed in future research. First, a cross-sectional design was used in this formative study to establish whether outdoor environments and experiences are associated with work outcomes, so an important next step would be to explore similar research questions using more advanced methods, such as longitudinal designs or experience sampling methodology (ESM). For example, a longitudinal study could assess the different places individuals work and live across their life course and examine how different locations influence experiences of the outdoors and work-related variables. In an ESM study, participants’ real-time locations could be reported or assessed using geographic data, while simultaneously assessing length of time outside, current levels of enjoyment, and activities engaged in, and then be examined alongside same- or next-day work outcomes. As previously mentioned, the effects detected in the present study are small. However, given the multitude of variables that can influence an employee’s engagement and creativity at work, it is not surprising that we found small effects related to the built and natural environment and experiences outside on these outcomes. Moreover, “moving the needle” even marginally on work-related outcomes is meaningful for both employees and organizations, particularly for modifications that can be low-cost and low-effort, like going outside. Conducting more rigorous studies using advanced designs may yield more precise effects that are stronger in magnitude.

In addition, the use of objective location data is a strength of the study because it is more reliable and unbiased than a participant’s perception of the degree of urbanity or natural amenities where they live and work. However, other variables were self-reported by participants (e.g., time spent outside, enjoyment of time outside, job engagement, creativity), introducing the possibility for common method bias (Podsakoff et al., 2012). Future research could employ the use of supervisor reports or customer satisfaction ratings as indicators of employees’ engagement and creativity (e.g., Wildman et al., 2011; Mumford and Todd, 2019). Another next step would be for researchers to further extend the conceptualization of contact with nature by exploring other features of the outdoor environment (e.g., tree canopy coverage, biodiversity; Keniger et al., 2013). Prior research has demonstrated the unique value of exposure to blue spaces (i.e., natural or human-made water features; lakes, ponds, fountains) as a promotive tool for improving health (Smith et al., 2021). Notably, exposure to combined blue-green spaces is a particularly beneficial coping tool during times of societal distress (e.g., COVID-19; Pouso et al., 2021), which is critical given over a quarter of Americans state that they are so stressed they cannot function, often citing societal stressors such as inflation, violence and crime, and political and racial climates (American Psychological Association [APA], 2022). Therefore, it may be worthwhile to explore nuances related to the degree of “greenness” and “blueness” of natural areas.

Additionally, the level of contact with nature at work is likely related to individuals’ jobs and industries, as jobs can vary according to the level of contact with nature from the job context and job tasks (Klotz and Bolino, 2021). For instance, warehouse forklift operators have low contact with nature at work in both their job context and job tasks, florists have high contact with nature in terms of job tasks but not job context, and farmers have high contact with nature in their job tasks and context (Klotz and Bolino, 2021). It may also be worthwhile to examine individuals whose job provides high levels of contact with green spaces (e.g., landscapers, botanists) compared to blue spaces (e.g., commercial divers, marine biologists). Future research could focus on a specific occupation or industry or examine job type as a substantive variable that may influence nature contact, the experience of time spent outside, and work outcomes.

Another potential methodological drawback is the combination of working students and participants collected on MTurk in the analyses. As previously noted, this decision was due to the students being co-located (thereby limiting the variance in the location-related predictor variables of urbanity and natural amenities), the need for a large enough sample size to have adequate power to detect indirect effects (Fritz and MacKinnon, 2007), and to account for potential season and COVID-related effects, as the data collections spanned the fall, winter, and spring months, and reflected the time period both before and during the COVID-19 pandemic (81.5% of the participants completed the survey before COVID). Although all participants reflected working adults, there were some differences between samples. Unsurprisingly, compared to participants in the MTurk sample, working students were younger, worked fewer hours, and were less likely to work a regular daytime schedule. Accordingly, we statistically accounted for the different samples as well as relevant characteristics (e.g., age). Additionally, we ran the mediation analyses with only the MTurk sample and found nearly identical results. In line with our previous recommendations, future work would benefit from examining employees working in the same type of job, rather than the broad approach we took in this study. Additionally, although not a focus of this study, it would also be interesting for future work to examine potential differences in outdoor experiences and related job outcomes prior to, compared to during, crises such as pandemics.

Alternative mechanisms that explain the associations found in the present study could be explored in future work. For example, it is possible that positive affect or emotions are acting as additional mediators, as nature can restore emotional energies (Klotz and Bolino, 2021), and having more leisure time and enjoying oneself can produce positive feelings (Newman et al., 2014), which can relate to greater engagement and creativity at work (Hennessey and Amabile, 2010; Bledow et al., 2011). Another possible explanatory mechanism is sleep, as sleep can be influenced by nature and time spent outside (Shin et al., 2020). American adults who live in areas with greater natural amenities and greenspace are more likely to report sufficient sleep (Grigsby-Toussaint et al., 2015) and have lower odds of short sleep (Johnson et al., 2018). Similar to contact with nature, healthy sleep is also critical for energy replenishment and related emotional, cognitive, and physical outcomes (e.g., Crain et al., 2018). Future research should also consider other aspects of workers’ health and well-being. For example, how does spending time outside during work and nonwork hours relate to job attitudes and the physical and mental health of employees? Is spending time outside or engaged in specific outdoor activities beneficial for reducing the deleterious effects of work stressors that are associated with poor mental health, or among workers with mental health conditions (e.g., ADHD and anxiety) (Follmer and Jones, 2018)?

As more research is being conducted that investigates issues related to climate change and work (e.g., Kjellstrom et al., 2016), understanding the interaction between work and engaging with nature is ripe for additional studies. For example, how might results vary based on climate in geographical regions that differ in terms of temperature, humidity, rate and types of natural disasters, or hours of sunlight? Are there differences across occupations that vary in the extent to which aspects of the work itself may be performed outside, and how might climate, and climate changes, relate to those experiences?

Researchers should consider other work outcomes in future studies. Klotz and Bolino (2021) theorize that contact with nature at work should also increase prosocial energy, which allows individuals to invest resources in the well-being of others. This is in line with the reasonable person model, which stipulates how supportive environments (which are often natural) enable reasonableness, cooperation, and helpfulness (Kaplan and Basu, 2015). These ideas about how nature can influence prosocial behaviors have gained some empirical support (e.g., Zhang et al., 2014; Zelenski et al., 2015). Individuals who have greater contact with nature at work and home may also exhibit more prosocial behaviors at work and fewer deviant behaviors at work. Examining other work-related variables is a promising avenue for future research.

## 5 Conclusion

We investigated the interplay among the built and natural environment at work and home (i.e., urbanity and natural amenities), experiences of the outdoor environment (i.e., time spent outside, enjoyment, activities), and work-related outcomes (i.e., job engagement and creativity). Overall, we found initial evidence that living and working in more natural areas is positively associated with spending time outside and enjoying time outside, which can replenish emotional, cognitive, and physical energies that enable employees to be engaged and creative at work.

## Data availability statement

The raw data supporting the conclusions of this article will be made available by the authors upon request, without undue reservation.

## Ethics statement

The studies involving humans were approved by Colorado State University Institutional Review Board. The studies were conducted in accordance with the local legislation and institutional requirements. The participants provided their written informed consent to participate in this study.

## Author contributions

RB: Conceptualization, Data curation, Formal analysis, Funding acquisition, Investigation, Methodology, Project administration, Supervision, Visualization, Writing – original draft, Writing – review & editing. TC: Conceptualization, Supervision, Writing – review & editing. JL: Data curation, Writing – review & editing. GF: Conceptualization, Supervision, Writing – review & editing. AE: Conceptualization, Supervision, Writing – review & editing.

## Footnotes

^1^All analyses were also performed without control variables. Without control variables included, the significant indirect effect of natural amenities at work on emotional job engagement via hours outside at work was not significant, as the 95% confidence interval included zero, though did not change sign. Additionally, without control variables included, the significant indirect effect of natural amenities at home on creativity via nonwork hours outside was not significant. Otherwise, all other significant indirect effects were retained.

^2^Given concerns about combining heterogeneous samples, we also performed all analyses using only participants from the MTurk sample (only the MTurk sample was adequately powered). In these analyses, the indirect effect of natural amenities at home predicting greater cognitive engagement via enjoyment of time spent outside was no longer significant. Otherwise, all other significant indirect effects were retained. Additionally, a new significant effect was detected in the MTurk-only analyses, such that there was a significant indirect effect of natural amenities at work on creativity via hours spent outside at work.

^3^Many responses included mentions of both intentional and incidental outdoor activities, so categorization was selected based on which type of activity was described to the greatest degree. Otherwise, responses in which participants described intentional and incidental outdoor activities to an equal extent (e.g., “walking either for pleasure or to a store”) were categorized in an intentional and incidental category.
